# A Novel Approach to Identifying Trajectories of Mobility Change in Older Adults

**DOI:** 10.1371/journal.pone.0169003

**Published:** 2016-12-22

**Authors:** Rachel E. Ward, Marla K. Beauchamp, Nancy K. Latham, Suzanne G. Leveille, Sanja Percac-Lima, Laura Kurlinski, Pengsheng Ni, Richard Goldstein, Alan M. Jette, Jonathan F. Bean

**Affiliations:** 1 New England GRECC, Boston VA Healthcare System, Boston, MA, United States of America; 2 Harvard Medical School, Cambridge, MA, United States of America; 3 Spaulding Rehabilitation Hospital, Cambridge, MA, United States of America; 4 School of Rehabilitation Science, McMaster University, Hamilton, Ontario, Canada; 5 Health and Disability Research Institute, Boston University School of Public Health, Boston, MA, United States of America; 6 College of Nursing and Health Sciences, University of Massachusetts Boston, Boston, MA, United States of America; 7 Massachusetts General Hospital, Boston, MA, United States of America; Iranian Institute for Health Sciences Research, ISLAMIC REPUBLIC OF IRAN

## Abstract

**Objectives:**

To validate trajectories of late-life mobility change using a novel approach designed to overcome the constraints of modest sample size and few follow-up time points.

**Methods:**

Using clinical reasoning and distribution-based methodology, we identified trajectories of mobility change (Late Life Function and Disability Instrument) across 2 years in 391 participants age ≥65 years from a prospective cohort study designed to identify modifiable impairments predictive of mobility in late-life. We validated our approach using model fit indices and comparing baseline mobility-related factors between trajectories.

**Results:**

Model fit indices confirmed that the optimal number of trajectories were between 4 and 6. Mobility-related factors varied across trajectories with the most unfavorable values in poor mobility trajectories and the most favorable in high mobility trajectories. These factors included leg strength, trunk extension endurance, knee flexion range of motion, limb velocity, physical performance measures, and the number and prevalence of medical conditions including osteoarthritis and back pain.

**Conclusions:**

Our findings support the validity of this approach and may facilitate the investigation of a broader scope of research questions within aging populations of varied sizes and traits.

## Introduction

Older adults tend to experience change in mobility and disability with great variation due to comorbidity and high rates of illness and injury. [1, 2] Longitudinal analyses examining disability progression have accounted for this variation by using multiple trajectories. [2–4] Often these analyses use advanced statistical techniques, such as latent class growth analysis or mixture modeling to identify distinct groups of individuals that follow similar patterns of change over time. However, this statistical methodology requires large sample sizes and several time points to ensure model convergence, validity, and sufficient statistical power. [5–7] Alternative methods that overcome these constraints would facilitate trajectory research among a wider variety of study designs and populations.

Most studies investigating disability trajectories in late-life have focused on activities of daily living (ADLs) and instrumental activities of daily living (IADLs), [2–4, 8, 9] and few have examined trajectories of mobility change. Mobility limitations, which conceptually precede disability within models of disablement, are typically the target of rehabilitative intervention. Limitations in mobility are common among older adults, affecting approximately one quarter of adults aged 70 years and older and half of those aged 80 years and older. [10] Mobility limitations pose a significant threat to the health and independence of older adults, leading to poor outcomes such as falling, hospitalization, nursing home admission and mortality. [11–17] The Boston Rehabilitative Impairment Study of the Elderly (Boston RISE), was designed to investigate key research questions identified by experts in geriatric rehabilitation, such as identifying underlying predictors of poor and declining mobility. [18] Thus, mobility trajectories represent a major outcome for Boston RISE.

While advanced statistical techniques used to model trajectories require adequate sample size and sufficient time points, the values for these requirements are not completely rigid and depend on additional factors such model complexity and the amount of variance explained by the model. [7] Nevertheless, there are certain generally accepted guidelines. For example, three or more follow-up time points are typically required, although two time points may be adequate in the case of partially missing data for some participants. [7, 19] In addition, a sample size of at least 100 participants is generally preferred for growth curve modeling; however the adequacy of this number is also dependent on the number of observations per individual, and therefore more participants may be needed when fewer follow-up time points are available. [7]

This study aimed to identify distinct trajectories of mobility change using 3 time points over 2 years of follow-up within 430 older adult primary care patients. To overcome the constraints of modest sample size and minimal follow-up time points, we generated trajectories of mobility change using a novel approach that incorporated both clinical reasoning and distribution-based methodology. We demonstrated the validity of our approach using latent class growth analysis and by comparing differences across trajectories in baseline mobility-related health conditions, body functions/impairments, personal factors, and physical function/activities consistent with the World Health Organization’s International Classification of Functioning, Disability, and Health and Nagi disablement models. [20–22] We hypothesized that that these factors, particularly body functions/impairments and physical function/activities, would differ across trajectories with more deficits observed for unfavorable mobility trajectories.

## Methods

The Boston Rehabilitative Impairment Study of the Elderly (Boston RISE) is a prospective cohort study designed to identify modifiable impairments that are associated with mobility in older adults. Study methods have been previously detailed. [23] Briefly, primary care patients aged ≥65 years were recruited from 9 practices across the greater Boston area from December 2009 to January 2012. Patients who met preliminary inclusion criteria within the practices were randomly selected for inclusion on a master recruitment list. The master list was divided into subgroups based on age, sex, and race, and oversampling was used to ensure that the cohort was representative of the older adult population residing within a 10 mile radius of the healthcare facility. Eligibility included difficulty or task modification with walking one-half mile and/or climbing 1 flight of stairs. [16] Exclusions included moderate or severe dementia (Mini-Mental State Examination score <18), and severe mobility limitation (Short Physical Performance Battery [SPPB] score <4). [24, 25] All methods were approved by the Spaulding Rehabilitation Hospital Institutional Review Board (approval number 2008P002472) and written consent was obtained from all participants. Baseline assessments were completed by 430 participants. This analysis included 391 participants (n = 8 died, n = 8 withdrew due to illness, n = 23 withdrew or were lost to follow-up) with mobility measured at baseline and either or both annual assessment in the two year follow-up period (n = 46 had outcome at year 1 but not year 2; n = 1 had outcome at year 2 but not year 1).

### LLFDI Lower-Extremity Function

The Lower-Extremity Function component of the patient-reported Late Life Function and Disability Instrument (LLFDI) is a widely used, validated measure of mobility [26] that assesses functional limitations applicable to daily life [27] consistent with both the International Classification of Functioning, Disability, and Health [20] and Nagi disablement [22] models. The LLFDI contains two lower-extremity scales: 1) Basic Lower-Extremity Function (BLE)–tasks involving standing, stooping, and basic walking; and 2) Advanced Lower-Extremity Function (ALE)–involving higher levels of physical ability and endurance, such as walking several blocks or standing up from the floor. Higher scores represent better mobility. We have previously shown that a change of 4.4 in BLE and 6.3 in ALE reflect the minimal detectable change with 90% confidence (MDC_90_). [28]

### Baseline Characteristics

This analysis included various baseline characteristics from several of the domains within the disablement models. Personal factors included age, sex, race, and education. Cognition was measured using the widely-used and validated Mini-Mental State Examination. [29, 30] Overweight and obesity status were defined using body mass index categories. Sensory loss was measured using the Semmes-Weinstein monofilament test. A 4.17 monofilament (providing a standardized force of 1.4g) was applied to the dorsum of the right toe until the thread buckled. If the participant could not feel the 4.17 monofilament during at least 3 out of 4 trials, a 5.07 monofilament (providing a standardized 10g force) was applied. Inability to feel both the 4.17 and the 5.07 monofilament during 3 out of 4 trials was considered evidence of sensory loss. [31] Several neuromuscular impairments that were associated with baseline mobility were included. [32] Strength was measured on a pneumatic leg press machine using a previously published protocol. [23] Strength asymmetry was calculated by dividing the higher value side by the lower value. Leg velocity was calculated by dividing peak leg press power by peak force. [33] Knee flexion and ankle range of motion (ROM) were measured using a goniometer. [34] Trunk extensor muscle endurance was measured with the participant lying prone on a specialized plinth positioned 45° from vertical using a previously published protocol. [35] Because some participants were unable to complete some of the neuromuscular tests (up to 14%), weighted multiple imputation was performed on these data. [36] The Activities-Specific Balance Confidence Scale is a valid and reliable measure of confidence in performing 16 daily activities without losing balance or becoming unsteady. [37, 38] Scores ranged from 0–100 with higher scores indicating higher confidence. The SPPB measured standing balance, usual paced walking speed, and 5-repetition chair stand time. Scores ranged from 0–12 with higher scores indicating better performance. Usual gait speed (m/s) was measured over 4 meters. A validated co-morbidity index measured the presence of 13 common chronic conditions. [39] Prevalence of the following individual conditions was also included: heart disease, hypertension, lung disease, diabetes, cancer, depression, osteoarthritis, back pain, neurologic disease, and peripheral arterial disease. Participants were asked if they had ever been told by a doctor or health professional that they had the disease.

### Statistical Analysis

Recent studies investigating disability among large cohorts of older adults have identified 5 distinct trajectories of change. [2, 3] Based on this literature and clinical reasoning, we generated a five level categorical variable to define persistent states and meaningful change in mobility for each the BLE and ALE scales of the LLFDI across baseline, year 1, and year 2. The five categories included: persistently poor, decline, persistently intermediate, improvement, and persistently high. “Decline” and “improvement” were defined as a decrease and increase, respectively, ≥MDC_90_ between any two time points. If both decline and improvement occurred, the amount of change from the first time point to the last was used as the tie breaker. When no meaningful change occurred, “persistently poor” and “persistently high” were defined as scores at the final follow-up (year 2 or 1) within the lowest or highest quartiles, respectively. “Persistently intermediate” was defined as scores at the last follow-up between the highest and lowest quartiles.

To validate the number of trajectories for both LLFDI scales, we performed latent class growth analysis, and estimated the log Bayes factor using the Bayesian Information Criterion (BIC) score with the following equation: 2 x [(BIC_greater trajectories model_)–(BIC_fewer trajectories model_)]; where a positive score indicates an increase in fit for the model with the greater number of trajectories and a negative score indicates a decrease in fit. [19, 40] This score is used for nested models, and is thus appropriate for testing the inclusion of different numbers of trajectories within a model. Guidelines for interpreting the extent of evidence provided by the log Bayes factor for model complexity while accounting for model parsimony are as follows: 0–2 = weak evidence; 2–6 = moderate evidence, 6–10 = strong evidence, and >10 = very strong evidence. [40] We additionally used model convergence to judge fit.

We evaluated known-groups validity among the trajectories by testing differences in baseline characteristics through three sets of pairwise comparisons: comparisons between the persistently poor and each of the other trajectories, comparisons between the persistently high and each of the other trajectories, and comparisons between the improvement and decline trajectories. Chi-squared, t-tests, and non-parametric equivalents were used with an alpha level of 0.01, since multiple comparisons were performed. We also performed two separate sensitivity analyses in which we adjusted comparisons for age and sex and tested the differences in neuromuscular impairments between the trajectories using non-imputed data.

## Results

When validating the number of trajectories generated for ALE mobility, we found positive log Bayes factor scores indicating an increase in fit for the models with the greater number of trajectories for 4 vs. 3 (log Bayes factor = 95.2) and 5 vs. 4 trajectories (log Bayes factor = 31.6), although the model with 5 trajectories did not converge. For BLE mobility, positive log Bayes factor scores indicated an increase in fit for the models with the greater number of trajectories for 4 vs. 3 (log Bayes factor = 32.5), 5 vs. 4 (log Bayes factor = 36.4), 6 vs. 5 (log Bayes factor = 41.2), and 7 vs. 6 trajectories (log Bayes factor = 11.3), although the model with 7 trajectories did not converge.

**Fig 1:**
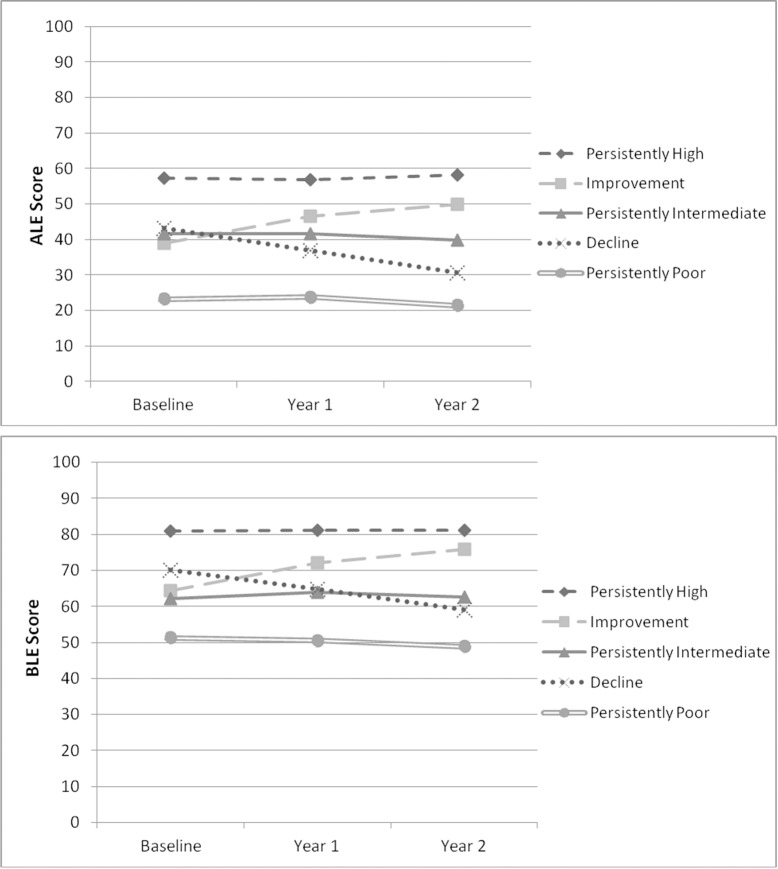
Mean mobility scores by ALE and BLE mobility trajectory from baseline through year 2 (N = 391). ALE = Advanced Lower-Extremity Function; BLE = Basic Lower-Extremity Function.

**Fig 2:**
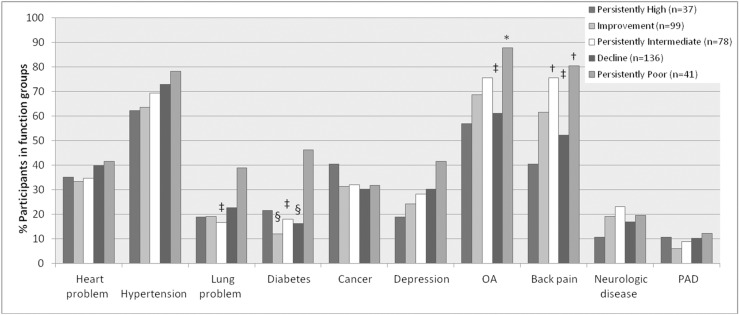
Baseline prevalence of self-reported chronic diseases by BLE mobility trajectory (N = 391). *p<0.01, †p<0.001 for comparisons with Persistently High. ‡p<0.01, §p<0.001 for comparisons with Persistently Poor. BLE = Basic Lower-Extremity Function; OA = Osteoarthritis; PAD = Peripheral Artery Disease.

Mean LLFDI scores for each trajectory at each time point are presented in Fig 1. Table 1 shows that participants with persistently poor ALE mobility were more likely to have a high school education, and less likely to have had graduate/professional schooling than those in the improvement and high mobility trajectories, although the latter were marginally significant (p<0.05). Participants with persistently high ALE mobility had the fewest chronic conditions, best knee flexion ROM and SPPB scores (Table 2), and a lower rate of back pain than those with persistently intermediate and persistently poor mobility (43.8% vs. 67.0% and 76.9%, respectively; p<0.01 for both). The persistently poor BLE trajectory had lower cognition scores (Table 3), the slowest limb velocity, poorest knee and ankle flexion ROM, the most chronic conditions (Table 4), and higher rates of lung problems, diabetes, osteoarthritis, and back pain (Fig 2). Both the persistently poor ALE and BLE mobility trajectories had the weakest leg strength and trunk extensor endurance, poorest balance confidence scores, worst SPPB scores, and slowest gait speed, while those in the persistently high mobility trajectories had the best leg strength, velocity, trunk extensor endurance, balance confidence scores, and gait speed (Tables 2 and 4). Findings from the sensitivity analyses in which we adjusted for age and sex and used non-imputed neuromuscular impairment data did not materially differ from the original results.

**Table 1:** Baseline characteristics by ALE mobility trajectory over 2 year follow-up (N = 391).

	Mean ± SD, Median (IQR), or n (%)				
Characteristics	Persistently High (n = 48)	Improvement (n = 68)	Persistently Intermediate (n = 115)	Decline (n = 134)	Persistently Poor (n = 33)
**Age, years**	74.0 (69.0–80.0)	73.0 (69.0–81.5)	76.0 (70.0–82.0)	77.0 (71.0–83.0)*	79.5 (72.0–84.0)
**Men, n (%)**	19 (37.3)	27 (39.7)	34 (29.6)	42 (31.3)	8 (30.8)
**White race, n (%)**	46 (90.2)	52 (76.5)	100 (87.0)	107 (79.9)	24 (92.3)
**Education**					
**Grade 11 or below, n (%)**	4 (7.8)	9 (13.2)	18 (15.7)	11 (8.2)	4 (15.4)
**High School, n (%)**	14 (27.5)	15 (22.1)‡	33 (28.7)	41 (30.6)	14 (53.9)*
**College, n (%)**	18 (35.3)	25 (36.8)	38 (33.0)	43 (32.1)	6 (23.1)
**Graduate/Professional, n (%)**	15 (29.4)	19 (27.9)	26 (22.6)	39 (29.1)	2 (7.7)
**MMSE (0–30)**	29.0 (26.5–30.0)	28.0 (26.0–29.0)	28.0 (26.0–29.0)	28.0 (27.0–29.0)	28.0 (26.0–29.0)
**Overweight, n (%)**	19 (37.3)	34 (50.8)	48 (41.7)	50 (37.3)	8 (30.8)
**Obese, n (%)**	16 (31.4)	20 (29.9)	41 (35.6)	49 (36.6)	13 (50.0)

*p<0.01 for comparisons with Persistently High.

‡p<0.01 for comparisons with Persistently Poor. ALE = Advanced Lower-Extremity Function; MMSE = Mini Mental State Exam.

**Table 2:** Baseline health and function by ALE mobility trajectory over 2 year follow-up (N = 391).

	Mean ± SD, Median (IQR), or n (%)				
Characteristics	Persistently High (n = 48)	Improvement (n = 68)	Persistently Intermediate (n = 115)	Decline (n = 134)	Persistently Poor (n = 33)
**Comorbidity index (0–13)**	3.0 (2.0–4.0)	4.0 (3.0–6.0)*	4.0 (3.0–5.0)	4.0 (3.0–5.0)†	5.0 (4.0–5.0)*
**Sensory loss, n (%)**	9 (18.8)	15 (22.1)	39 (34.8)	41 (31.3)	9 (36.0)
**Strength (N/kg)**	10.4 (9.0–13.4)	9.8 (8.0–11.2)§	9.0 (7.6–10.5)†,‡	9.3 (7.2–10.8)†,‡	7.1 (6.5–9.4)†
**Strength asymmetry (ratio)**	1.08 (1.04–1.16)	1.16 (1.06–1.29)	1.09 (1.03–1.21)	1.12 (1.05–1.22)	1.11 (1.07–1.31)
**Trunk endurance (s)**	150.0 (150.0–150.0)	122.3 (47.0–150.0)†,‡	150.0 (55.0–150.0)†,§	90.9 (30.1–150.0)†,‡	45.0 (0.0–78.9)†
**Limb velocity (m/s)**	1.09 ± 0.20	1.00 ± 0.27	1.00 ± 0.25	0.96 ± 0.27*	0.90 ± 0.23†
**Knee flexion (deg)**	131.0 (124.0–138.5)	125.0 (117.0–133.5)*	126.0 (120.0–131.0)*	126.0 (115.0–134.0)*	120.0 (114.0–124.0)†
**Ankle flexion ROM Impaired, n (%)**	5 (10.4)	17 (25.0)	30 (26.3)	47 (35.1)*	10 (38.5)*
**ABC (0–100)**	90.6 (85.0–93.8)	79.1 (67.2–90.0)†,§	78.1 (66.9–88.1)†,§	76.3 (65.0–88.8)†,§	65.9 (51.3–71.9)†
**SPPB score (0–12)**	10.5 (9.0–11.5)	9.5 (7.0–11.0)*,§	9.0 (8.0–10.0)†,§	9.0 (7.0–10.0)†,‡	7.0 (5.0–9.0)†
**Usual gait speed (m/s)**	1.04 ± 0.17	0.90 ± 0.25†	0.94 ± 0.19*,‡	0.88 ± 0.21†	0.81 ± 0.20†

*p<0.01

†p<0.001 for comparisons with Persistently High.

‡p<0.01

§p<0.001 for comparisons with Persistently Poor.

ALE = Advanced Lower-Extremity Function; ROM = range of motion; ABC = Activities-Specific Balance Confidence; SPPB = Short Physical Performance Battery.

**Table 3:** Baseline characteristics by BLE mobility trajectory over 2 year follow-up (N = 391).

	Mean ± SD, Median (IQR), or n (%)				
Characteristics	Persistently High (n = 37)	Improvement (n = 99)	Persistently Intermediate (n = 78)	Decline (n = 136)	Persistently Poor (n = 41)
**Age, years**	74.0 (70.0–81.0)	76.0 (70.0–81.0)	76.0 (71.0–82.0)	76.0 (71.0–83.0)	75.0 (69.0–81.0)
**Men, n (%)**	19 (39.6)	30 (30.3)	29 (37.2)	48 (35.3)	7 (17.1)
**White race, n (%)**	42 (87.5)	76 (76.8)	67 (85.9)	120 (88.2)	30 (73.17)
**Education**					
**Grade 11 or below, n (%)**	4 (8.3)	10 (10.1)	8 (10.3)	17 (12.5)	6 (14.63)
**High School, n (%)**	10 (20.8)	23 (23.2)	21 (26.9)	42 (30.9)	18 (43.9)
**College, n (%)**	18 (37.5)	42 (42.4)	29 (37.2)	40 (29.4)	10 (24.4)
**Graduate/Professional, n (%)**	16 (33.3)	24 (24.2)	20 (25.6)	37 (27.2)	7 (17.1)
**MMSE (0–30)**	29 (28–30)	28 (26–29)	28 (27–29)	28 (27–29)	27 (25–29)*
**Overweight, n (%)**	16 (33.3)	47 (48.0)	33 (42.3)	49 (36.0)	10 (24.4)
**Obese, n (%)**	17 (35.4)	29 (29.6)	29 (37.2)	50 (36.8)	21 (51.2)

*p<0.01 for comparisons with Persistently High. BLE = Basic Lower-Extremity Function; MMSE = Mini Mental State Exam.

**Table 4:** Baseline health and function by BLE mobility trajectory over 2 year follow-up (N = 391).

	Mean ± SD, Median (IQR), or n (%)				
Characteristics	Persistently High (n = 37)	Improvement (n = 99)	Persistently Intermediate (n = 78)	Decline (n = 136)	Persistently Poor (n = 41)
**Comorbidity index (0–13)**	4.0 (2.0–5.0)	4.0 (3.0–5.0)§	4.0 (3.0–5.0)‡	4.0 (2.5–5.0)§	5.0 (4.0–7.0)†
**Sensory loss, n (%)**	10 (27.0)	20 (21.1)	24 (30.8)	43 (32.3)	16 (39.0)
**Strength (N/kg)**	10.7 (8.8–12.6)	9.7 (7.9–11.2)§	9.1 (7.4–10.6)*,‡	9.5 (8.0–10.9)*,§	7.2 (6.1–9.0)†
**Strength asymmetry (ratio)**	1.10 (1.06–1.20)	1.11 (1.04–1.25)	1.12 (1.05–1.25)	1.10 (1.04–1.19)	1.18 (1.06–1.39)
**Trunk endurance (s)**	150.0 (150.0–150.0)	150.0 (53.9–150.0)§	104.1 (47.0–150.0)*	121.2 (41.6–150.0)*,‡	56.6 (6.8–98.4)†
**Limb velocity (m/s)**	1.14 ± 0.22	1.00 ± 0.23*,§	1.01 ± 0.25§	1.00 ± 0.27*,§	0.81 ± 0.23†
**Knee flexion ROM (deg)**	129.0 (121.0–138.0)	129.0 (122.0–135.0)§	125.5 (116.0–133.0)§	125.0 (118.0–132.0)§	116.5 (111.0–124.0)†
**Ankle flexion ROM Impaired, n (%)**	10 (27.0)	23 (23.5)‡	21 (26.9)	35 (25.7)‡	20 (48.8)
**ABC Scale (0–100)**	91.3 (85.3–95.0)	80.0 (68.8–90.6)†,§	75.0 (66.3–85.3)†,§	83.3 (68.5–90.3)†,§	54.1 (42.8–69.7)†
**SPPB score (0–12)**	10.0 (9.0–11.0)	10.0 (8.0–11.0)§	9.0 (7.0–11.0)§	9.0 (7.0–10.0)§	7.0 (5.0–9.0)†
**Usual gait speed (m/s)**	1.06 ± 0.20	0.94 ± 0.24*,§	0.91 ± 0.19†,§	0.91 ± 0.19†,§	0.75 ± 0.19†

*p<0.01

†p<0.001 for comparisons with Persistently High.

‡p<0.01

§p<0.001 for comparisons with Persistently Poor.

BLE = Basic Lower-Extremity Function. ROM = range of motion; ABC = Activities-Specific Balance Confidence; SPPB = Short Physical Performance Battery.

## Discussion

Findings from this study demonstrate the feasibility and validity of a novel approach for identifying mobility trajectories within a modest sized cohort over 2 years. This approach extends the use of trajectory analyses to studies with smaller sample sizes and fewer follow-up time points than previously allowed by traditional statistical approaches. Such findings have the potential to promote the investigation of a broader scope of research questions involving trajectory analyses within aging populations of varied sizes and traits.

Model fit indices showed very strong evidence (log Bayes factor >10) that the optimal number of trajectories fell between 4 and 6, suggesting our selection of 5 trajectories was appropriate. This number of trajectories is also consistent with the number reported in previous studies of disability among large cohorts of older adults. [2, 3] Although we used a clinically intuitive approach, we observed similar patterns of mobility change to those previously described using advanced statistical techniques. For example, the decline trajectory described in this analysis, is conceptually similar to “progressive”, “developing”, [2] or “accelerated increase” [3] in disability trajectories from other studies. However, less often included is an “improvement” or “recovery” trajectory, [41] despite high rates of recovery from disability reported in older adults. [42] We found that 25% and 17% of our cohort experienced meaningful improvement in BLE and ALE mobility, respectively. It is possible that we were able to detect improvement in mobility while others were not due to responsiveness of the LLFDI to improvement, demonstrated previously within this study population. [28] Categorizing individuals with improvement in mobility is particularly important for research informing interventions since it supports that improvement of mobility is achievable for older adults.

Overall, we found that participants in the persistently poor mobility trajectories had the least favorable mobility-related baseline characteristics, while participants in the persistently high mobility trajectories had the most favorable characteristics. This included a number of personal, health-related, body function/impairment, and physical function/activity factors. The improvement and decline in mobility trajectories had more favorable characteristics than the poor mobility trajectories and less favorable characteristics than the high mobility trajectories; however, the characteristics of the improvement and decline trajectories did not differ from each other. This may be evidence of contributing factors to change in mobility that were not included in this analysis, such as healthcare leading to improvement in mobility or medical complications leading to decline.

This is the first study to identify mobility trajectories using the LLFDI, a widely used, validated measure of mobility, specifically designed to be responsive to change. [26] We previously showed within this cohort, that the LLFDI detected meaningful decline and improvement comparably to performance-based measures such as the SPPB and gait speed. [28] This analysis extends these results by showing that the LLFDI is able to capture a number of different trajectories of meaningful mobility change over a relatively short time period of 2 years.

### Limitations

Although targeted recruitment within this study resulted in demographic distributions consistent with the 2004 census for older adults living within the recruitment area, [32] our findings may not be fully generalizable to older adults within other geographical regions. Some participants were unable to perform some neuromuscular tests resulting in missing data; to address this we performed multiple weighted imputation on these data. [36] Persistently poor and high mobility trajectories had modest numbers of participants, which may have resulted in low power for some comparisons. Despite this, we found numerous significant differences when comparing these trajectories to others. We only assessed baseline predictors of mobility and the duration of our follow-up was relatively short; however, this may mirror annual wellness visits in which a clinician must target risk factors for mobility decline assessed within a single initial evaluation. Future work should examine the predictive ability of these different trajectories on key health-related outcomes. Further validation of this approach may be warranted using additional instruments.

### Conclusion

Using a novel approach designed to overcome constraints of modest sample size and few follow-up time points, we identified five trajectories of mobility change within this study of older adults. We demonstrated the validity of this approach by using latent class growth analysis to confirm the optimal number of trajectories and by comparing known mobility-related characteristics between the trajectories. This approach has the potential to extend trajectory analysis to a wider variety of studies and populations.
